# Instability in CH_3_NH_3_PbI_3_ perovskite solar cells due to elemental migration and chemical composition changes

**DOI:** 10.1038/s41598-017-15841-4

**Published:** 2017-11-13

**Authors:** Zubair Ahmad, Mansoor Ani Najeeb, R. A. Shakoor, Abdulla Alashraf, Shaheen A. Al-Muhtaseb, Ahmed Soliman, M. K. Nazeeruddin

**Affiliations:** 1 Center for Advanced Materials (CAM), Qatar University, P.O.Box 2713 Doha, Qatar; 2 Department of Chemical Engineering, College of Engineering, Qatar University, P.O.Box 2713 Doha, Qatar; 3 Gas Processing Center (GPC), Qatar University, P.O.Box 2713 Doha, Qatar; 4 Group for Molecular Engineering of Functional Materials, Ecole Polytechnique Fédérale de Lausanne (EPFL), CH-1015 Sion Lausanne, Switzerland

## Abstract

Organic-inorganic halide perovskites have rapidly grown as favorable materials for photovoltaic applications, but accomplishing long-term stability is still a major research problem. This work demonstrates a new insight on instability and degradation factors in CH_3_NH_3_PbI_3_ perovskite solar cells aging with time in open air. X-ray photoelectron spectroscopy (XPS) has been used to investigate the compositional changes caused by device degradation over the period of 1000 hrs. XPS spectra confirm the migration of metallic ions from the bottom electrode (ITO) as a key factor causing the chemical composition change in the perovskite layer besides the diffusion of oxygen. XPS results are in good agreement with the crystallographic marks. Glow discharge optical emission spectrometry (GD-OES) has also been performed on the samples to correlate the XPS results. Based on the experimental results, fundamental features that account for the instability in the perovskite solar cell is discussed.

## Introduction

Chemical instability of organic-inorganic hybrid lead halide perovskites limits their performance and durability in various applications such as solar cells^1,2^, photo-assisted water splitting^3,4^, solid-oxide fuel cells^5,6^ etc. Even though the progress of perovskite solar cells (PSCs) has gone from operating under unstable liquid electrolytes far to solid state hole-transporting materials (HTMs)^7^, a proper understanding of degradation mechanisms for perovskite materials and their relevant solutions still need to be explored. Various environmental factors including moisture, ultraviolet light and thermal stress, play a key role in the instability of perovskite materials^8–10^. When exposed to moisture, perovskite materials tend to hydrolyze, which results in their disintegration back into their precursors and finally irretrievable degradation of the perovskite structures. So far, several solutions have been proposed to make the perovskite materials more stable. For instance, by the assistance of cross-linking additives, material instability can be addressed to a certain extent^11^. Further, compositional engineering^12,13^ and the use of cation cascade technique^14^ has been recently demonstrated to reduce the material photo-instability. Efforts have also been put forth to alleviate the effect of moisture and other factors on the stability of perovskite solar cells by partial substitution of I with Br ions^15^, device architecture^16,17^, coating the perovskite cells with a water-proof fluorinated polymer^18^ and deposition methods^19^.

Michael Grätzel *et al*.^20^, reported that replacing iodide by bromide in the mixed perovskite results in a blue shift of the absorption edge, which makes these mixed cation/anion perovskites outstandingly more stable under photo-illumination. *Zhang et al*.^21^ incorporated CuPc nanorods as a hole-selective contact material, together with the printable carbon as a cathode material, considerably high power conversion efficiency (PCE) and improved durability relative to doped-spiro-OMeTAD/ based device was reported. *Kang, et al*.^22^, reported a water-repellent perovskite structure based on anti-reflective graded pyramidal arrays that were made-up using polydimethylsiloxane (PDMS) film that confirmed excellent hydrophobicity and made the PSC water-repellent. *Lee et al*.^23^ recommended crystal chemistry engineering as one of the optimistic methods for enhancing perovskite stability. The introduction of a dopant-free hole transporting material for perovskite solar cells by Rakstys *et al*.^24^ demonstrated good thermal, electrochemical and photochemical stability. Nazeeruddin’s group also synthesized molecularly engineered novel dopant-free star-shaped D-π-A type hole transporting materials, which in combination with mixed-perovskite (FAPbI_3_)0.85(MAPbBr_3_)0.15 (MA: CH_3_NH_3_^+^, FA: NH = CHNH_3_^+^) exhibit an excellent power conversion efficiency (PCE) of 18.9% under AM 1.5 conditions. The PSC based on FA-CN showed an exceptional stability up to 500 hrs.

Perovskite solar cells are heterogeneous systems comprising of materials with different morphologies and physical/chemical characteristics. Typical Perovskite solar cell structures consist of indium tin oxide (ITO)/ fluorine doped tin oxide (FTO), TiO_2_ (compact and mesoporous) layers followed by the perovskite layer, hole transport layer and the top metal electrode. The diffusion of the top gold electrode into the perovskite layer has already been reported by Matteocci *et al*.^25^. The indium and tin from the ITO layer can migrate towards the perovskite layer when exposed to moisture. Indium, tin and titanium diffusion towards the perovskite absorber layer could also be a reason for degradation in the perovskite solar cells. However, a deep analysis is required to investigate chemical changes due to the elemental migration effect from the ITO/FTO layer to the perovskite layer.

In this work, to attain a comprehensive evidence about the chemical state and chemical composition of the perovskite layers, x-ray photoelectron spectroscopy (XPS) analyses of the CH_3_NH_3_PbI_3_ perovskite films have been performed. CH_3_NH_3_PbI_3_ perovskite solar cells with best PCE of 17% has been reported by *Im, J.-H., et al*.^26^ We used the XPS technique to quantify the CH_3_NH_3_PbI_3_ perovskite ingredients and to compare between fresh and aged samples. We have found that the elemental migration and chemical changes due to oxygen element could be among the major factors that cause instability in CH_3_NH_3_PbI_3_ perovskite solar cells.

## Results

**Figure 1:**
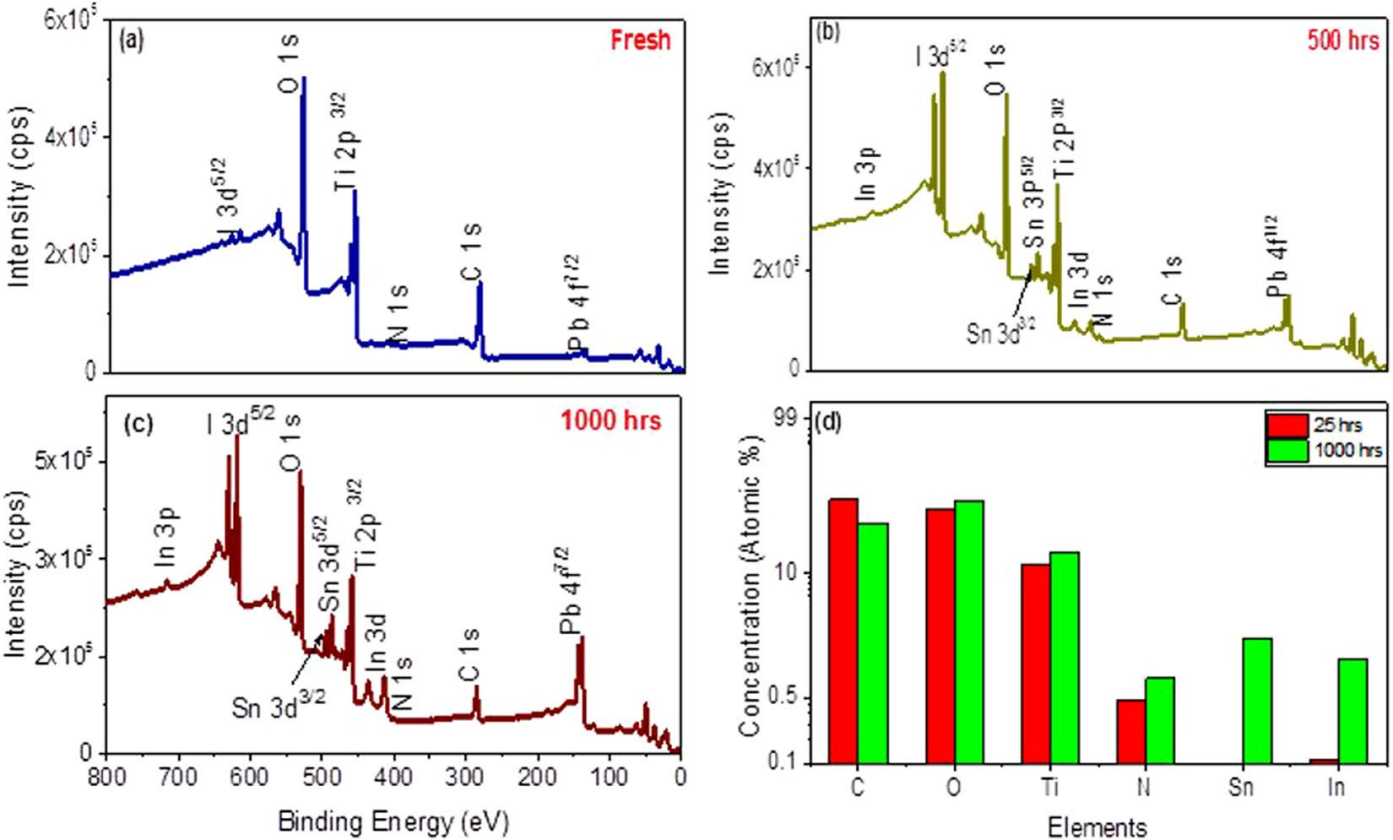
XPS survey spectra of the perovskite films documented for (**a**) fresh samples (taken after 25 hrs of sample preparation) and after the aging of (**b**) 500 hrs and (**c**) 1000 hrs, in open air under the room temperature. The graph (**d**) shows a comparison of the concentrations (in atomic %) of C, N, O, Ti, In and Sn between fresh and aged (1000 hrs) perovskite layer.

To determine the elemental composition of the perovskite absorber layers, XPS survey spectra were recorded at different time spans in the binding energy range of 0 to 800 eV. XPS measurements with probe depths of up to10 nm were performed. The XPS survey spectra for fresh (taken after 25 hrs of the sample preparation) and aged samples (after 500 hrs and 1000 hrs) are given in Fig. 1. The identified elements in the samples are indium, tin, titanium, lead, iodine, oxygen, nitrogen and carbon. The presence of carbon, nitrogen, lead and iodine are predictable from the chemical composition of the CH_3_NH_3_PbI_3_ perovskite film. However, the indium, tin, titanium peaks are resulting due to the migration of these elements from the ITO and TiO_2_ layers towards the top layer with the passage of time. Figure 1d shows a comparison of the elemental concentrations in perovskite films for freshly prepared and 1000 hrs aged sample. The elemental concentration data are taken from the XPS survey spectra given in Fig. 1a and c. It has been found that a very negligible (0.11 atomic %) concentration of indium is present in freshly prepared samples, whereas, the tin concentration was almost nil. Nevertheless, the indium and tin concentrations reached to 2.1 and 1.3 atomic %, respectively, after 1000 hrs of aging. Carbon contents are found to be reduced from 47.6% to 30.3% in 1000 hrs. There is no significant increase recorded in oxygen contents in first 500 hrs. However, it increased from 39.3 atomic %, (freshly prepared) to 41.1 atomic %, after aging for 1000 hrs in ambient air. A slight rise in the Ti content (from 15.80 to 16.16 atomic %) can also be noticed after the aging of 1000 hrs. Perovskite materials are well known to have great tendency to absorb moisture. Nevertheless, in the case of CH_3_NH_3_PbI_3_, there is a minor variation of oxygen contents during the 1000 hrs of aging. However, the major cause of chemical instability has been found due to the migration of tin and indium into the absorber layer.

**Figure 2:**
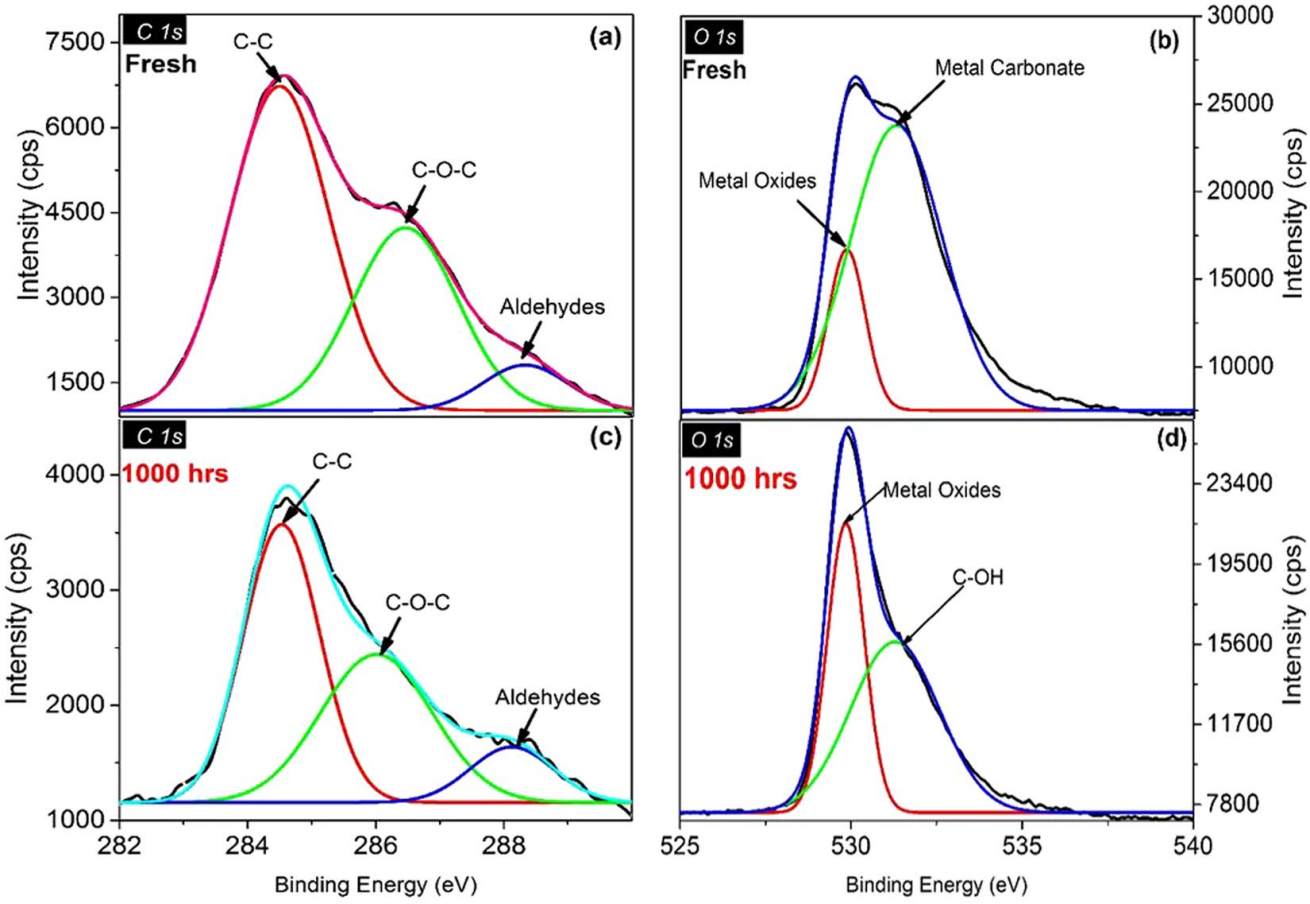
Core level XPS spectra (C 1 s and O 1 s) for fresh (**a** and **b**) and 1000 hrs aged (**c** and **d**) samples. C 1 s indicates the broadening of the high energy peak. Whereas, O 1 s spectra show that the metal carbonate turned to the carboxyl group due to chemical reaction.

**Figure 3:**
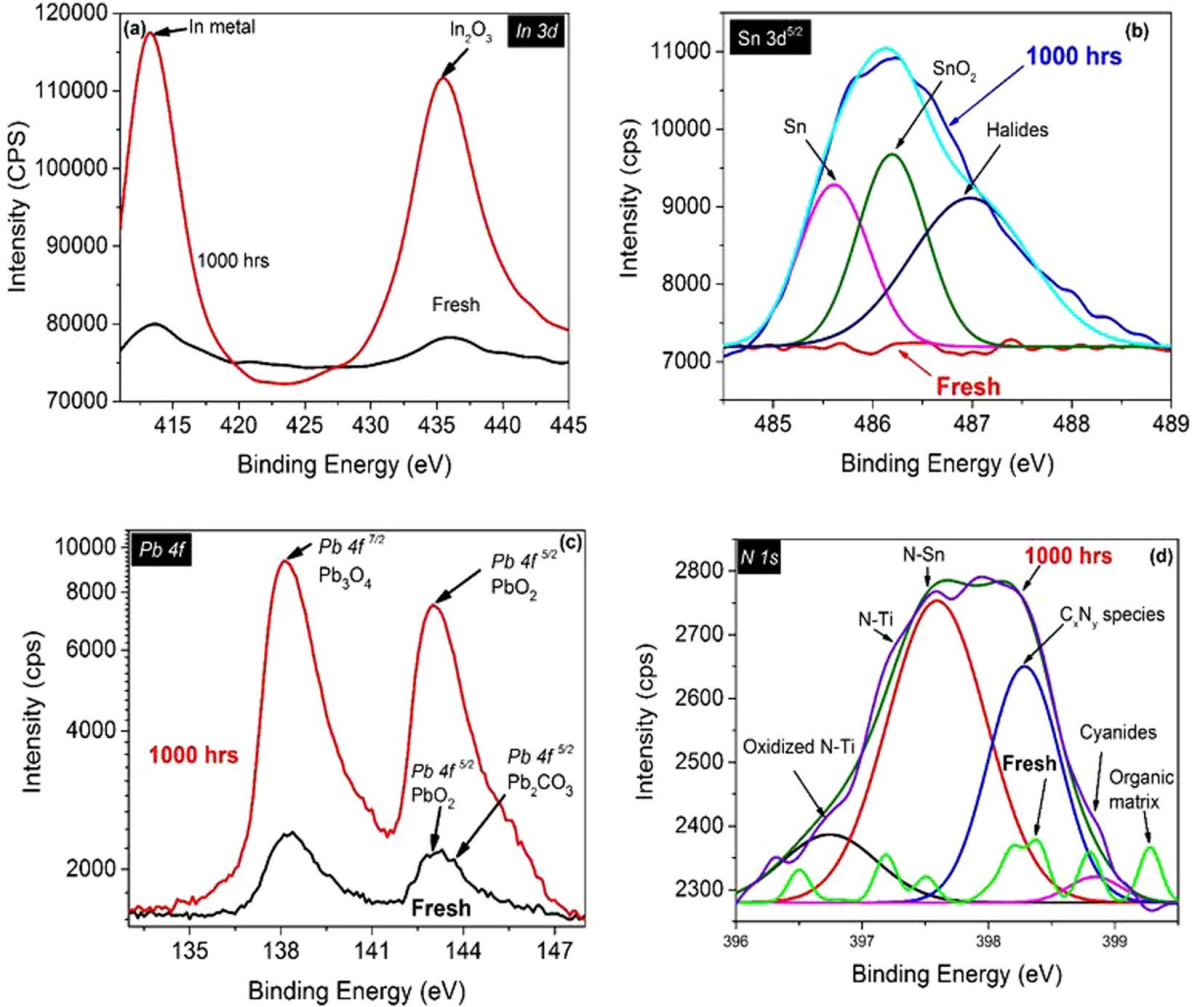
Core level XPS spectra (of (**a**) In 3d, (**b**) Sn 3d^5/2^, (**c**) Pb 4 f and (**d**) N 1 s) for fresh sample and 1000 hrs aged samples. The In 3d spectra indicates the significant increase of the In metal contents and formation of the In_2_O_3_ at the surface of the perovskite layer after the aging of 1000 hrs. Sn 3d^5/2^ spectra show the existence of the Sn after 100 hrs. Also, the formation of the SnO_2_ and halides is very clear in the aged sample. Pb 4 f spectra clearly indicates that the PbO_3_ and PbO_2_ have been significantly increased in the aged sample. N 1 s spectra exhibit the formation of metal nitrides in the aged sample.

High resolution XPS core spectra were undertaken to elucidate changes in chemical bonding in CH_3_NH_3_PbI_3_ films due to the diffusion of indium and tin. C 1 s, N 1 s, O 1 s, In 3d, Sn 3d, Pb 4 f, I3d and Ti 2p core-level spectra of perovskite films show the new sighting about the elemental migration and chemical changes that occurred in the perovskite layer. Figure 2 shows the C 1 s and O 1 s spectra on the surface of the CH_3_NH_3_PbI_3_ films. The intensity of carbon peaks has significantly reduced after ageing as shown in the C 1 s spectra for fresh and aged samples (Fig. 3a and b, respectively), which is also apparent in the survey spectra (Fig. 2). This points to the reduction in carbon contents in the perovskite layer. In C 1 s spectrum for the fresh sample, the components at 284.6 and 286.3 and 288.3 eV refer, respectively, to C-C bond, C-O bond and aldehydes. The peak at 284.6 eV is consistent to the sp3 carbon^27^. The change in term of broadening in the C 1 s spectrum towards the higher binding energy has been observed with the passage of time. The 288.3 eV peaks in the C 1 s spectra have been shifted to 288.4 eV after 1000 hrs of aging. The high binding energy broadening in the aged samples specifies the growth of oxygenated carbon in the perovskite layer. The high energy values at 288.3 and 288.4 eV for the fresh and 1000 hrs aged samples, respectively, are well matched with the carbon singly or doubly bonded to oxygen, i.e., O-C**=**O or C-O-C bonds. Conversely, the C=O and C-O oxidation of the carbon atoms cannot be distinguished due to the widening of the peak. The oxidization of carbon atoms might be one of the reasons that cause the degradation in the performance of perovskite materials in the solar cells. The O 1 s spectra before and after ageing are given in Fig. 3c and d, respectively. The components at 529.9 and 531.0 eV in the fresh sample assign to metal oxide and metal carbonate. After aging the sample for 1000 hrs, the high energy peak O1s has been shifted (approx. 0.8 eV) in the direction of higher binding energy. In the O 1 s spectrum of the aged sample a new peak at 531.79 eV (which corresponds to the carboxyl group) appeared instead of 531.0 eV (which corresponds to metal carbonate).

To study the chemical changes due to the diffusion of the indium and tin into the perovskite absorber layer, high-resolution XPS spectra of these components has been recorded as shown in Fig. 3a and b. The spectrum of the indium exhibits two gears. The crests at the 413.2 and 435.2 eV correspond to indium metal and In_2_O_3_, respectively. The 3d^5/2^ core spectrum of the tin displays three components at the binding energies of 485.6, 486.2 and 487 eV, which correspond to tin, tin oxide, and halide, respectively. The counts per second (CPS) intensity of the tin on the surface of the sample is very low as compared to the indium because of its lesser concentration in ITO as compared to the indium. The moisture absorbed by the perovskite film plays a leading role to accelerate the migration of indium and tin from the ITO layer, where the migration rate depends upon the moisture level in the air and the exposure period. The influence of the moisture level and exposure time on the migration rate must be studied separately.

The core level spectrum of lead (Fig. 3c) shows two distinct peaks at Pb 4f^5/2^ (approximately 143 eV) and Pb 4f^7/2^ (138 eV). A close look on the high-energy peak of the fresh sample indicated the existence of the PbO_2_ and Pb_2_CO_3_. PbCO_3_ is presented at around 143.8, whereas PbO_2_ is represented at around 143 eV. Nonetheless, after 1000 hrs of aging, the peak at 143 eV becomes very dominant, which indicates the formation of PbO_2_. Lead perhaps turned to PbO_2_ and Pb_2_CO_3_ on the surface of perovskite layer through its chemical reaction with oxygen and carbon dioxide, respectively. The multiplex N1s XPS spectrum (Fig. 3d) of perovskite layer shows several distinct peaks. The N1s spectrum of the 1000 hrs aged sample depicts four distinct peaks at 396.8, 397.17, 398, and 398.8 eV. It also indicated the formation of two major components with different chemical structure, i.e., metal nitrides and cyanides. The metal nitrides are in the range of 397.3 ± 1.1 eV. Three peaks of the metal nitrides at 396.8, 397.2 and 397.17 eV have been identified as N-Pb, N-Ti, and N-Sn, whereas the peak at 398 eV represents the C_x_N_y_ species^28^. The peak allocation is consistent with published literature, and identical tendencies have been documented for other metal nitrides as well. The peak at 398.8 eV refers to the cyanides. In the case of the fresh sample, the peak of the organic matrix can be clearly seen. However, this peak disappeared in the aged samples.

**Figure 4:**
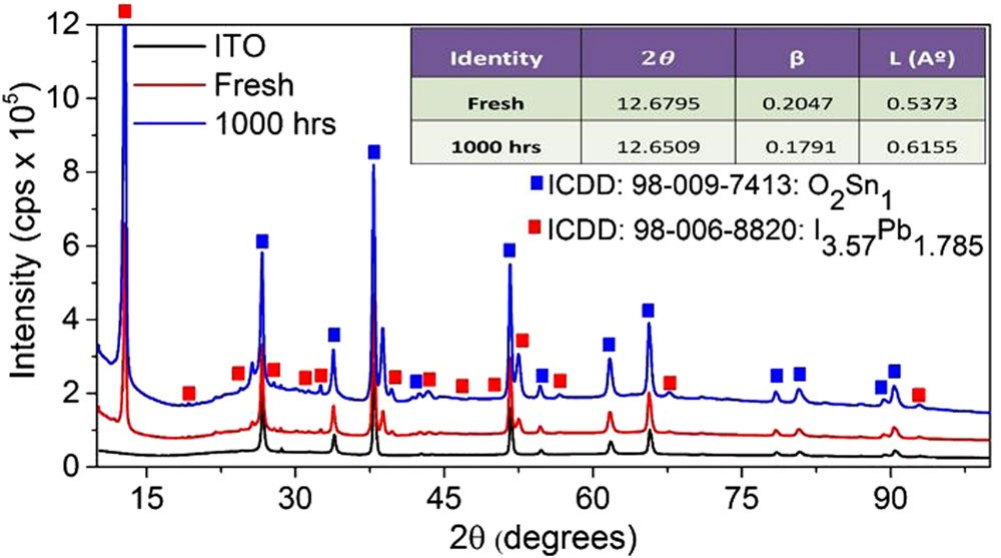
XRD spectra for the ITO film on glass substrate, fresh sample and 1000 hrs aged sample. The table given in the inset shows that line boarding decreased and grain size is increased in the aged sample.

In the fresh sample, the iodine 3d peak (Fig. 4a) contains a single peak at 629.6 eV, which can be endorsed to the presence of iodide. Though, after aging for 1000 hrs, the intensity of the 629.6 eV peak is reduced; and an additional peak appears at 622.6 eV, which can be referred to the oxidized iodine. The results are in good agreement with the XRD study as shown in Fig. 4 (and data files are given in supplementary data file S). The XRD spectra of the ITO, fresh and 1000 hrs aged samples. It has been noticed that a crystalline structure perseveres in the both fresh and aged samples. However, the peaks of the aged sample demons a minor shift in XRD 2*θ* position (as shown in the table in the inset of Fig. 4). The change in the 2*θ* value towards the lower value in the aged sample suggests the expansion of unit cells. The grain size (*L*) of the fresh and aged samples has been calculated using the following expression^29^:

1 $$L=\frac{K\lambda }{\beta \,(2\theta )\times \,\mathrm{Cos}\,\theta }$$

where, *β* represents the line broadening at FWHM, *K* is a shape-factor (dimensionless). The value of shape factor is supposed to be 0.9 (close to unity), *θ* is the Bragg angle (in degrees), *λ* is the X-ray wavelength, and *L* is the mean size of the crystalline domains. The calculated parameters presented in the table evidently shows that the aging affects the mean size of the crystalline domains of CH_3_NH_3_PbI_3_. The increase in L gives a clear hint that there is a considerable increase in the perovskite crystals size.

**Figure 5:**
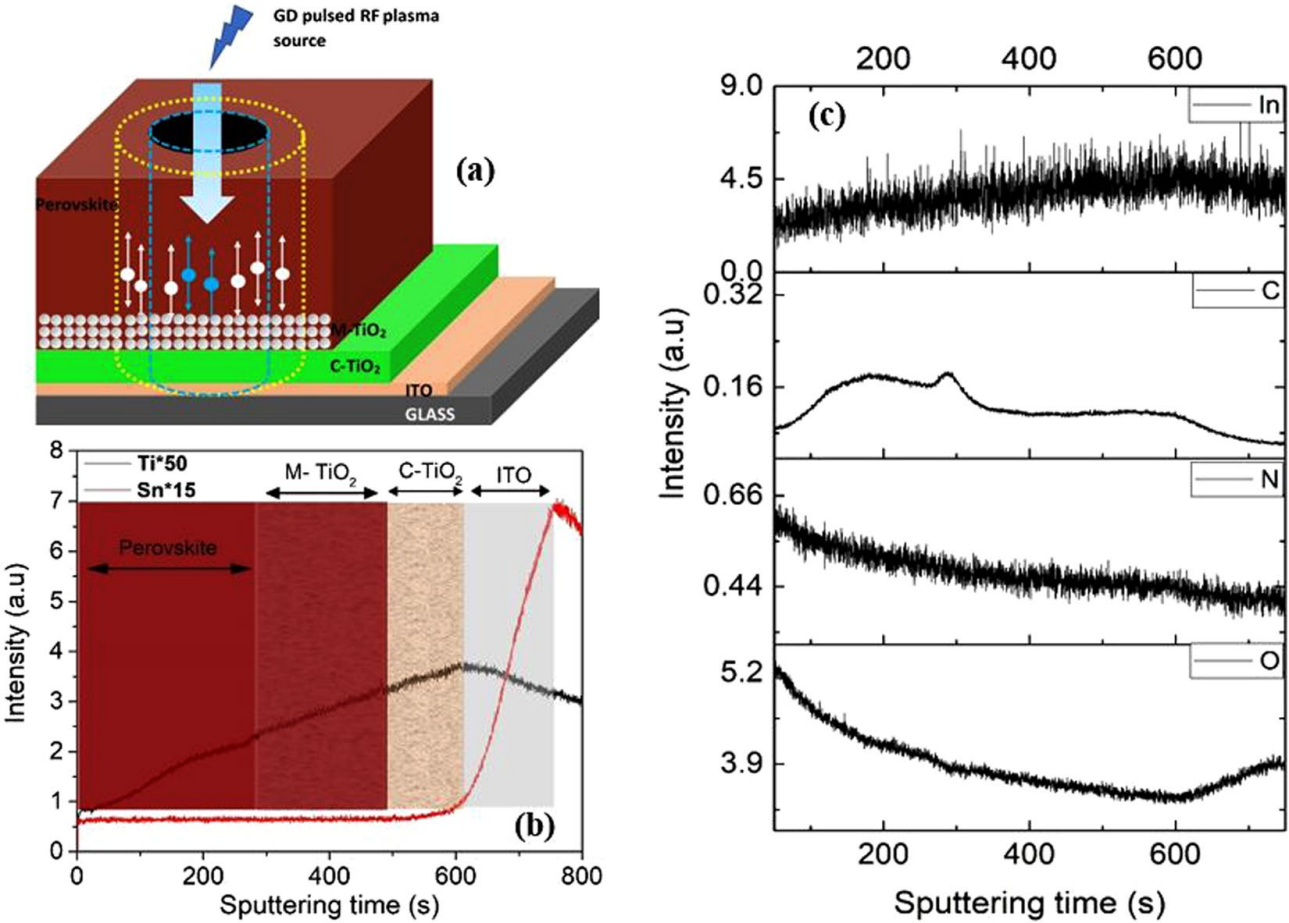
(**a**) Schematic diagram of the device architecture used for GD-OES analysis. GD-OES elemental profile (**b**) for Ti and Sn (**c**) for In, C, N and O.

In order to correlate the XPS results, glow discharge optical emission spectrometry (GD-OES) has also been performed on the samples, the results are reported in Fig. 5. GD-OES is a spectro-chemical technique which allows the direct trace of the major elements^30^. Figure 5a shows the scheme of the sample used for the GD-OES profile analysis. During the GD-OES experiment, the sputtering starts from the surface of the sample (perovskite layer) to the ITO layer (as shown in Fig. 5a. Thus, the GD-OES signals reveal consist of the perovskite layer, mesoporous-TiO2 (M-Tio2), compact-TiO2 (C-TiO2) and ITO. The profile was stopped once the sputtering reached the ITO layer. Figure 5b and c presents the elemental distribution of indium (In), carbon (C), oxygen (O), nitrogen (N) Titanium (Ti) and Tin (Sn), which are the main elements that have been investigated by the XPS analysis as well. It is worth noting that the existence of the In and Tin can be clearly seen near the surface of the sample in the GD-OES profile (Fig. 5b and c). In Fig. 5b the GD-OES profile is referred to the relative atomic composition of the Ti and Sn and the different layers (corresponding to the elemental mapping) have been marked as shown in the Figure. It is important to note that the intensities of the GD-OES signal for each element (in the Fig. 5b and c) vary in large amplitude. However, these relative intensities do not correspond directly to the relative concentrations due to the different sputtering rates of each layer and different emission yields for each element^30^.

## Discussion

The results given above provide the elemental analysis and chemical compositional changes on the top surface of the CH_3_NH_3_PbI_3_ perovskite absorber layer. In the case of a typical perovskite solar cell device structure, the absorber layer is separated by the transparent conductive electrode (TCE) by the compact TiO_2_ layer, which may offer poor blockade against the diffusion of indium and tin. Thus, perovskite absorber layer is significantly affected by the indium and tin diffusion, which might be a cause to initiate the instability in the perovskite solar cells. However, it is known that the perovskites are protected by HTMs. HTM layer acts as a protective layer over the perovskite material from the air and inhibits the diffusion of exterior moieties or elements inside the absorber. It also fills the porosity of the perovskite absorber layer. If the samples were analyzed without HTM, then there will be question the validity of your experiments.

Therefore, the samples were also compared with HTM coated CH_3_NH_3_PbI_3_. To investigate the effect of HTM, the most commonly used HTM (spiro-OMeTAD) was used. The layer of spiro-MeOTAD is deposited over the perovskite crystal pellets using spin coating. The XPS spectra of the aged samples with HTM layer is given in supplementary data file S. It is supposed that it cannot investigate the perovskite film by XPS if coated with the HTM, therefore, XPS spectra of the HTM coated perovskite was performed on the top surface of the perovskite layer after the *in-situ* etching of the HTM layer using argon ion gun. Figure S4 and S5 (in the supplementary data file S), still clearly reveals the migration of the elements and diffusion of the oxygen into the perovskite layer. Even though the spiro-MeOTAD protected the diffusion of the oxygen up to some extend but it is not strong enough to counteract the penetration of elements and safeguard to chemical changes. This might be due to the presence of “pin-holes” in the HTL, which leads to the permeation of oxygen and moisture in to the active layer. Furthermore, the samples were also prepared using fluorine tin oxide (FTO) as a TCE instead of the ITO. Clear migration of tin and florine also found in this case as well as shown in the Figure S6 in supplementary file.

In summary, XPS analyses show the compositional and chemical changes within the perovskite layer in open air. The penetration of indium and tin towards the top surface of perovskite layer is found. Further, the oxidation of carbon, nitrogen and the formation of the metal nitride have been evidently observed. A significant increase in the tin and indium contents perovskite surface upon the aging of 1000 hrs indicate that the diffusion of these elements could be a major reason for the instability of perovskite solar cells. It can be concluded that the perovskite has been strongly affected by chemical reactions occurring in the perovskite layer due to the penetration of indium, tin from ITO/FTO surface rather than the oxidation. Based on our findings we suggest to enhancing the stability of perovskite solar cells by depositing a blocking layer by Atomic Layer Deposition (ADL) that should have strong resistance against the diffusion of indium and tin.

## Methods

**Figure 6:**
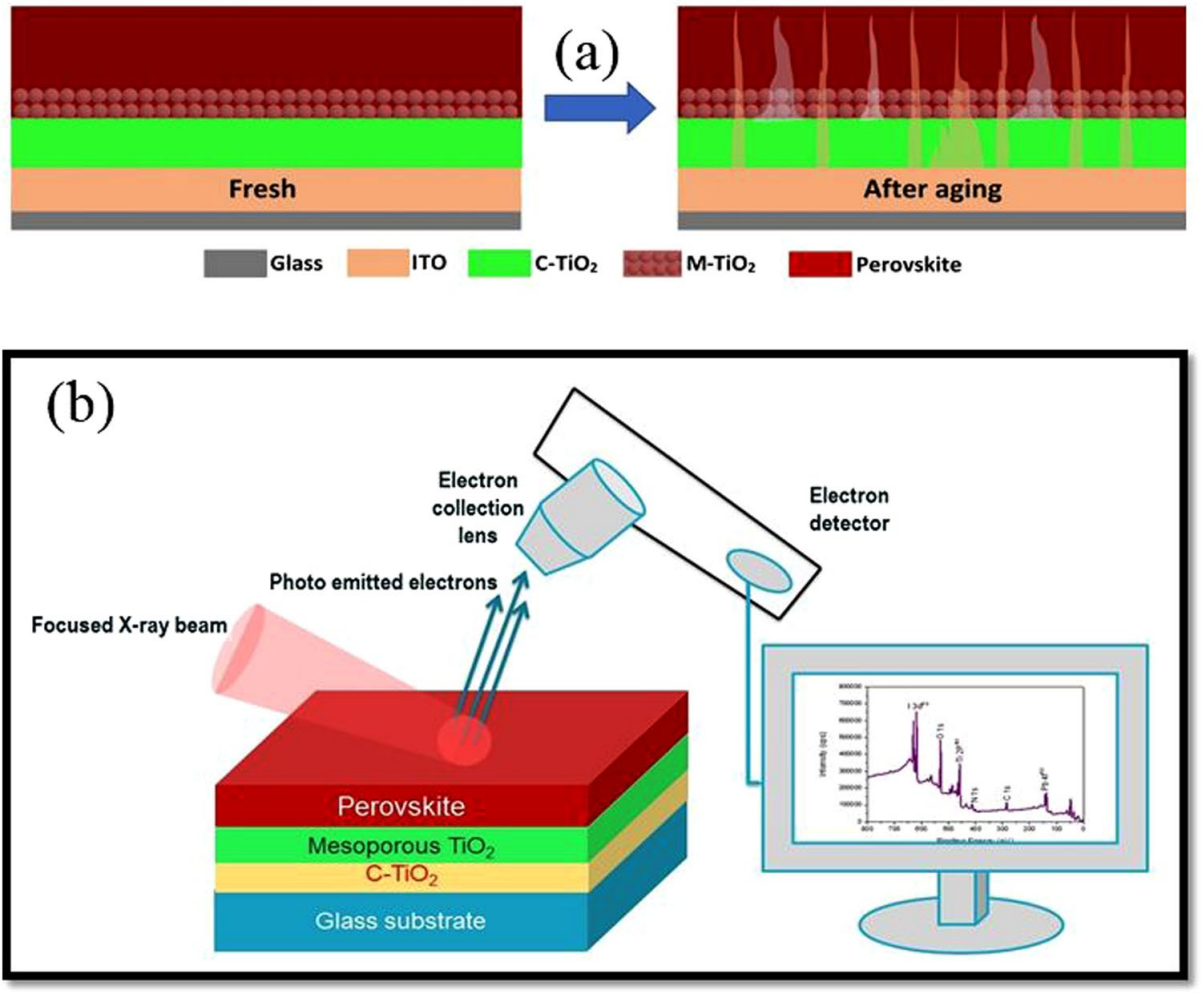
(**a**) Schematic diagram of the samples prepared for XPS analyses. The samples consist of ITO substrates followed by the deposition of compact and mesoporous titanium dioxide (TiO_2_) nanoparticles layers. The CH_3_NH_3_PbI_3_ perovskite layer was deposited on the top of the TiO_2_ mesoporous layer. The aged sample shows the diffusion of the indium and tin after 100 hrs. Figure (**b**) illustrates the XPS working procedure.

Perovskite layers for the XPS study were fabricated by following the previously reported procedure by *Im, J.-H., et al*.^26^. Briefly, ITO substrates were cleaned in an ultrasonic bath containing acetone, ethanol and distilled water for 20 min. TiO_2_ solution, for blocking layer, was prepared using 0.15 M titanium diisopropoxide bis (acetylacetonate) (75 wt% in isopropanol, Aldrich) mixed in 1-butanol (99.8%, Aldrich) solution. This solution was spin-coated on ITO substrates at 3,000 r.p.m. for 30 s and was heated at 150 °C for 5 min to get a ∼50 nm thick film. Once the blocking layer is dried, TiO_2_ paste (Dyesol) was applied over it using “Doctor Blade Technique” to a thickness of approximately ∼150 nm. The film was then sintered at 450 °C for 30 minutes. For the preparation of CH_3_NH_3_PbI_3_ perovskite layer (∼200 nm), a two-step spin coating procedure is used. 1 M of PbI_2_ solution was prepared by dissolving 462 mg PbI_2_ (99%, Aldrich) in 1 ml N,N-dimethylformamide (DMF, 99.8%, Sigma-Aldrich) under stirring at 70 °C. Before returning to room temperature, this solution was spin coated on the mesoporous TiO_2_ layer at a speed of 2,000 r.p.m. for 10 s. Without any loading time, the second layer of PbI_2_ solution was again spin coated at 5,000 r.p.m. for 10 s and dried at 50 °C for 5 min. 200 µl of 0.038 M CH_3_NH_3_I solution was deposited on the PbI_2_-coated substrate with a loading time of 30 s and spin rate of 3,000 r.p.m, and then dried at 100 °C for 5 min. Elemental analysis, chemical bonding and identification of the oxidation states were studied by XPS. Axis Ultra DLD (XPS) provides quantitative chemical state information up to10 nm of the surface depth, was used. Figure 6a shows the schematic diagram of the samples (fresh and aged) for the investigation whereas, Fig. 6b shows the schematic diagram of the XPS working procedure. GD-OES studies were performed using a GD Profiler 2 from HORIBA Jobin Yvon. For the GD-OES analysis, the selected size of the GD-OES spot was 4 mm in diameter, whereas the source was used in pulsed mode operation (power 17 W, pressure 420 Pa, pulse frequency 3 kHz, duty cycle 0.25).

## Electronic supplementary material

Supplementary Information
