# Supplementary material for: Instability in CH3NH3PbI3 perovskite solar cells due to elemental migration and chemical composition changes
Source: Sci Rep. 2017 Nov 13;7:15406. doi: 10.1038/s41598-017-15841-4 (PMC5684397; doi:10.1038/s41598-017-15841-4)

Instability in $CH_3NH_3PbI_3$ perovskite solar cells due to elemental migration and chemical composition changes

Zubair Ahmad[a]*, Mansoor Ani Najeeb[a], R.A. Shakoor[a], Abdulla Alashraf[a], Shaheen A. Al-Muhtaseb[b] Ahmed Soliman[c] and M. K. Nazeeruddin[d]

[a] Center for Advanced Materials (CAM), Qatar University, P.O.Box 2713, Doha, Qatar.
[b] Department of Chemical Engineering, College of Engineering, Qatar University, P.O.Box 2713, Doha, Qatar
[c] Gas Processing Center (GPC), Qatar University, P.O.Box 2713, Doha, Qatar.
[d] Group for Molecular Engineering of Functional Materials, Ecole Polytechnique Fédérale de Lausanne (EPFL), CH-1951 Sion, Switzerland

*Corresponding authors: zubairtarar@qu.edu.qa,

Ph: +974 4403 7729

Figure S1: I and O chemical composition in Fresh sample

No.	Ref. Code	S	Compound Na...	Chemical Formula	Scale ...	ML	NML	TL	SUL	Displac...	Q	Subfil...	Cryst. Syst.
1	ICSD 98-003-7059	16	Dioxygen	O2	0.031	3	3	15	0	0.000	=	User I...	Orthorhombic
2	ICSD 98-019-0368	15	Oxygen	O2	0.182	4	4	13	0	0.000	=	User I...	Hexagonal
3	ICSD 98-019-0369	15	Oxygen	O2	0.244	4	4	13	0	0.000	=	User I...	Hexagonal
4	ICSD 98-001-5535	11	Oxygen - Beta	O2	0.064	4	4	14	0	0.000	=	User I...	Hexagonal
5	ICSD 98-017-3934	9	Oxygen - Beta	O2	0.022	3	3	16	1	0.000	=	User I...	Hexagonal
6	ICSD 98-002-1107	5	Oxygen - Gamma	O2	0.040	5	5	17	1	0.000	=	User I...	Cubic
7	ICSD 98-001-0084	5	Diiodine	I2	0.017	7	7	40	0	0.000	=	User I...	Orthorhombic
8	ICSD 98-005-1500	4	Ozone	O3	0.350	28	28	1...	1	0.000	=	User I...	Orthorhombic
9	ICSD 98-017-3933	4	Oxygen - Alpha	O2	0.077	4	4	32	1	0.000	=	User I...	Monoclinic
10	ICSD 98-002-7516	4	Iodine(V) Oxide	I2 O5	0.031	30	30	1...	0	0.000	=	User I...	Monoclinic
11	ICSD 98-007-8387	4	Diiodine Penta...	I2 O5	0.031	30	30	1...	0	0.000	=	User I...	Monoclinic
12	ICSD 98-023-6858	4	Oxygen - Eta P...	O8	0.025	9	9	54	1	0.000	=	User I...	Monoclinic
13	ICSD 98-002-9064	4	Ice Ic	H2 O1	0.003	4	4	8	1	0.000	=	User I...	Cubic
14	ICSD 98-023-6855	3	Oxygen - Eta P...	O8	0.011	11	11	51	1	0.000	=	User I...	Monoclinic
15	ICSD 98-018-2673	3	Iodine Oxide (2...	I2 O6	0.330	27	27	1...	1	0.000	=	User I...	Anorthic
16	ICSD 98-023-6856	3	Oxygen - Eta P...	O8	0.010	10	10	49	1	0.000	=	User I...	Monoclinic
17	ICSD 98-003-0602	2	Hydrogenpero...	H2 O2	0.254	5	5	40	1	0.000	=	User I...	Tetragonal
18	ICSD 98-001-6950	2	Ice VI	H2 O1	0.017	8	8	51	1	0.000	=	User I...	Tetragonal
19	ICSD 98-002-8530	2	Samarium Oxid...	Br1 O1 Sm1	0.149	8	8	30	1	0.000	=	User I...	Tetragonal
20	ICSD 98-006-7618	2	Iodine	I2	0.018	11	11	66	1	0.000	=	User I...	Orthorhombic
21	ICSD 98-010-9040	1	Iodine - Iii, Hp	I1	0.097	1	1	9	1	0.000	=	User I...	Tetragonal
22	ICSD 98-009-2775	1	Oxygen	O1	0.310	8	8	46	1	0.000	=	User I...	Orthorhombic
23	ICSD 98-001-6817	1	Ice III	H2 O1	0.096	5	5	32	1	0.000	=	User I...	Tetragonal
24	ICSD 98-016-4724	0	Oxygen - Eta	O2	1.015	1	1	13	1	0.000	=	User I...	Hexagonal
25	ICSD 98-009-9593	0	Indium Chromi...	Cr0.333 In1 O3.333...	0.032	4	4	30	1	0.000	=	User I...	Monoclinic

Figure S2: I and O chemical composition in 1000 hrs aged sample

No.	Ref. Code	S	Compound Na...	Chemical Formula	Scale ...	ML	NML	TL	SUL	Displac...	Q	Subfil...	Cryst. Syst.
7	ICSD 98-018-2670	7	Iodine Oxide (2...	I2 O4	0.131	47	47	1...	1	0.000	=	User I...	Monoclinic
8	ICSD 98-002-7878	6	Ice Ic	H2 O1	0.004	3	3	8	1	0.000	=	User I...	Cubic
9	ICSD 98-002-9064	6	Ice Ic	H2 O1	0.005	4	4	8	1	0.000	=	User I...	Cubic
10	ICSD 98-002-9066	6	Ice Ic	H2 O1	0.004	4	4	8	1	0.000	=	User I...	Cubic
11	ICSD 98-007-8903	5	Tetraiodine Do...	I4 O12	0.112	57	57	1...	1	0.000	=	User I...	Anorthic
12	ICSD 98-008-0196	5	Diiodine(V) Dii...	I4 O12	0.112	57	57	1...	1	0.000	=	User I...	Anorthic
13	ICSD 98-005-1500	5	Ozone	O3	0.186	29	29	1...	1	0.000	=	User I...	Orthorhombic
14	ICSD 98-023-6857	5	Oxygen - Eta P...	O8	0.035	17	17	52	1	0.000	=	User I...	Monoclinic
15	ICSD 98-017-3934	4	Oxygen - Beta	O2	0.013	4	4	16	1	0.000	=	User I...	Hexagonal
16	ICSD 98-002-7877	4	Ice Ic	H2 O1	0.004	3	3	8	1	0.000	=	User I...	Cubic
17	ICSD 98-018-2673	4	Iodine Oxide (2...	I2 O6	0.280	32	32	1...	1	0.000	=	User I...	Anorthic
18	ICSD 98-001-0084	4	Diiodine	I2	0.019	5	5	40	0	0.000	=	User I...	Orthorhombic
19	ICSD 98-001-5398	4	Periodic Acid	H5 I1 O6	0.017	44	44	1...	1	0.000	=	User I...	Monoclinic
20	ICSD 98-002-4073	3	Hydrogenpero...	H6 O4	0.013	29	29	1...	1	0.000	=	User I...	Monoclinic
21	ICSD 98-001-6950	3	Ice VI	H2 O1	0.003	9	9	51	0	0.000	=	User I...	Tetragonal
22	ICSD 98-003-0602	3	Hydrogenpero...	H2 O2	0.290	6	6	40	1	0.000	=	User I...	Tetragonal
23	ICSD 98-004-3430	3	Oxygen - Beta	O2	0.008	5	5	16	1	0.000	=	User I...	Hexagonal
24	ICSD 98-001-6817	2	Ice III	H2 O1	0.028	5	5	32	1	0.000	=	User I...	Tetragonal
25	ICSD 98-009-2775	2	Oxygen	O1	0.114	12	12	46	1	0.000	=	User I...	Orthorhombic
26	ICSD 98-002-7516	2	Iodine(V) Oxide	I2 O5	0.042	32	32	1...	1	0.000	=	User I...	Monoclinic
27	ICSD 98-002-8530	1	Samarium Oxid...	Br1 O1 Sm1	0.178	8	8	30	1	0.000	=	User I...	Tetragonal
28	ICSD 98-010-9040	1	Iodine - Iii, Hp	I1	0.080	1	1	9	1	0.000	=	User I...	Tetragonal
29	ICSD 98-007-8387	1	Diiodine Penta...	I2 O5	0.041	32	32	1...	1	0.000	=	User I...	Monoclinic
30	ICSD 98-009-9593	1	Indium Chromi...	Cr0.333 In1 O3.333...	0.017	7	7	30	1	0.000	=	User I...	Monoclinic
31	ICSD 98-015-1434	1	Iodine - V	I1	0.113	1	1	14	1	0.000	=	User I...	Orthorhombic
32	ICSD 98-020-1246	1	Silver Iodide - ...	Ag1 I1	1.448	1	1	10	1	0.000	=	User I...	Cubic

Figure S3: XRD patterns with assigned crystal planes of the perovskite phases.

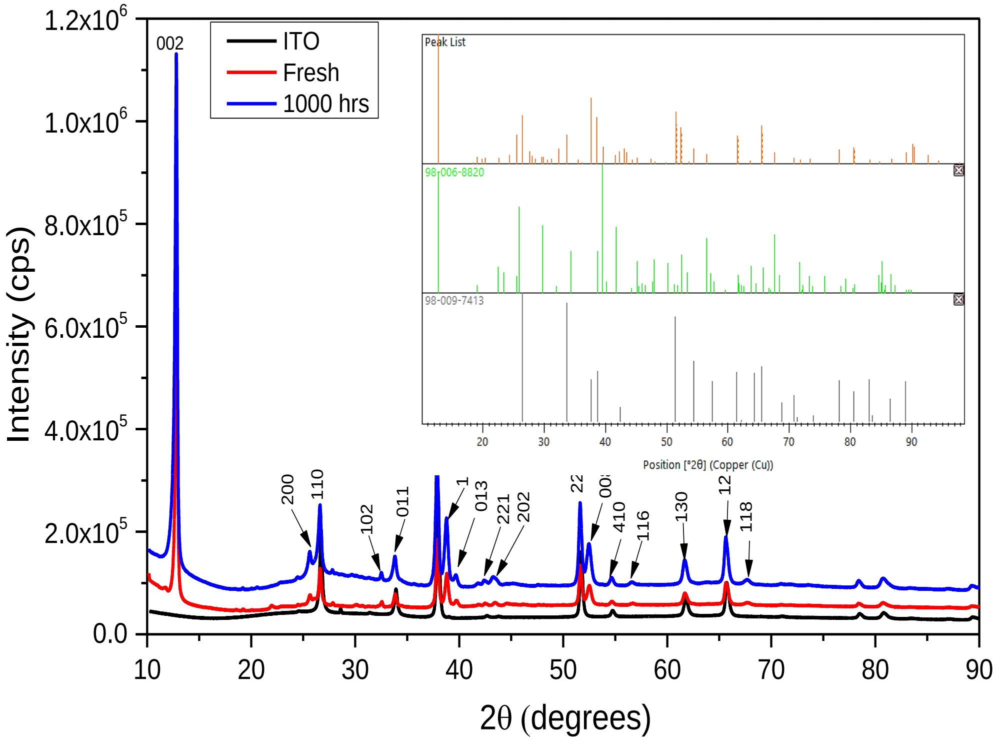

Figure S4: XPS survey spectrum of the ITO/C-TiO2/M-Tio2/ $CH_3NH_3PbI_3$/ HTM (spiro-OMeTAD) sample (after 100 hrs of preparation).

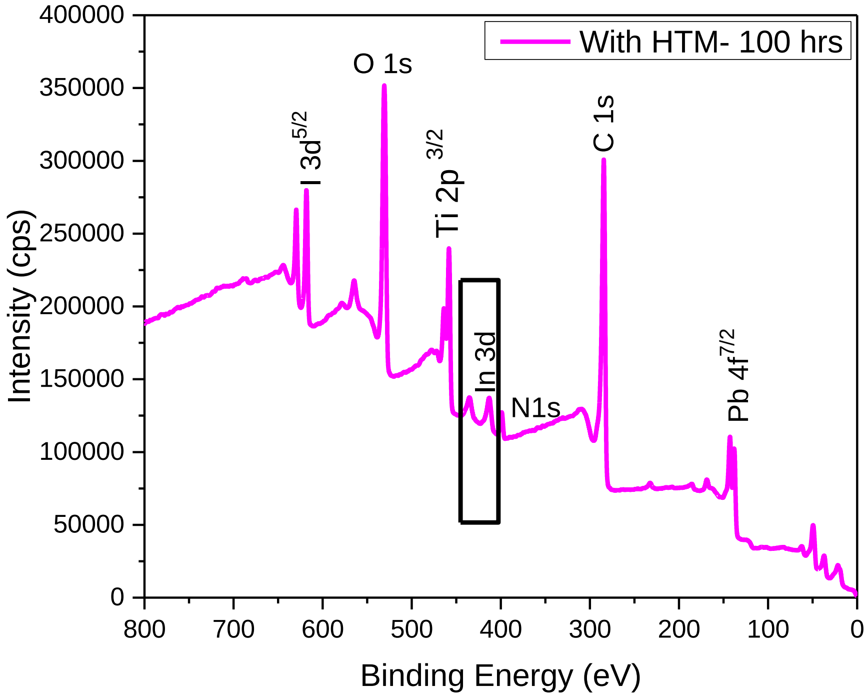

Figure S5: XPS survey spectrum of the ITO/C-TiO2/M-Tio2/ $CH_3NH_3PbI_3$/ HTM (spiro-OMeTAD) sample (after 750 hrs of aging).

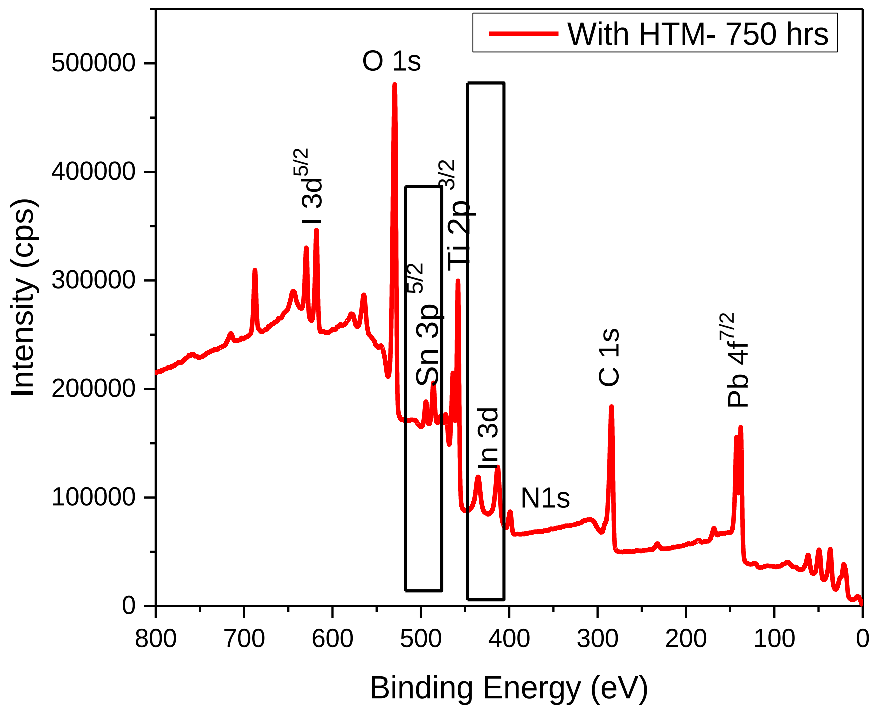

Figure S6: XPS survey spectrum of the FTO/C-TiO2/M-Tio2/ $CH_3NH_3PbI_3$/ HTM (spiro-OMeTAD) sample (after 1000hrs of preparation).

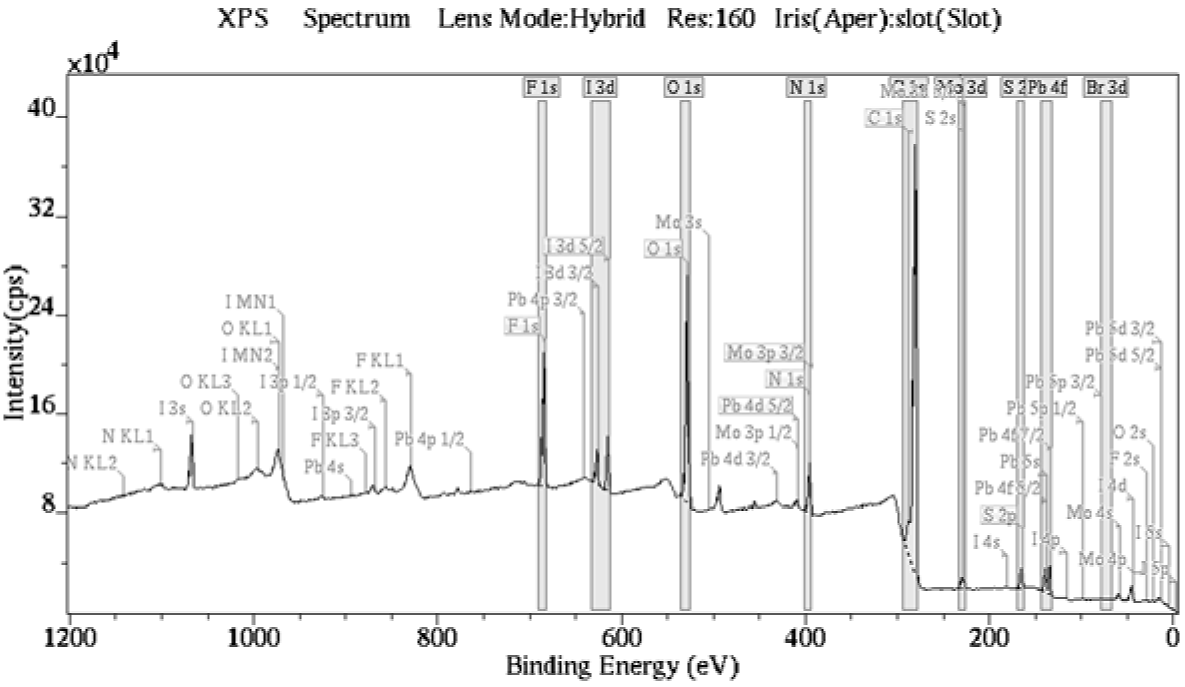

Supplement: Supplementary file 1 — Supplementary Information [file 41598_2017_15841_MOESM1_ESM.pdf]
